# Supplementary material for: High Expression of CUL9 Is Prognostic and Predictive for Adjuvant Chemotherapy in High-Risk Stage II and Stage III Colon Cancer
Source: Cancers (Basel). 2022 Aug 9;14(16):3843. doi: 10.3390/cancers14163843 (PMC9406063; doi:10.3390/cancers14163843)
Supplement: Supplementary file 1 [file cancers-14-03843-s001.zip › cancers-1849817-supplementary.pdf]

cancers

# Supplementary Material: High Expression of CUL9 Is Prognostic and Predictive for Adjuvant Chemotherapy in High-Risk Stage II and Stage III Colon Cancer

**Peng Zheng, Yang Lv, Yihao Mao, Feifan Shen, Zhiyuan Zhang, Jiang Chang, Shanchao Yu, Meiling Ji, Qingyang Feng, Jianmin Xu**

## Implementation of chemotherapy

In the clinical practice of our center, adjuvant chemotherapy was routinely recommend to high-risk stage II and stage III patients according to NCCN guidelines. Optional chemotherapy regimens includes FOLFOX and CapOX. The specific choice depends on the doctor. In case of intolerance of chemotherapy, we usually try a dose reduction by around 20%-25% first. If still intolerant, we will try no more dose reduction and stop chemotherapy.

In this study, the three chemotherapy groups are mainly identified according to the number of finished cycles, and the dose reduction is taken into account when calculating the cycles. A finished cycle is identified as a cycle with full dose or only one dose reduction by around 20%-25%. Details of adjuvant chemotherapy were summarized in Table S4.

A

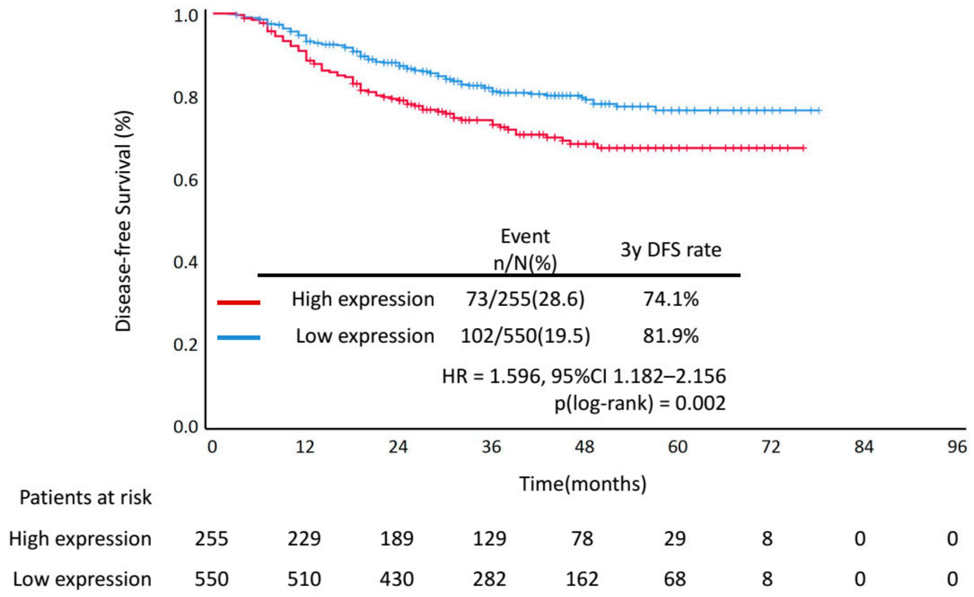

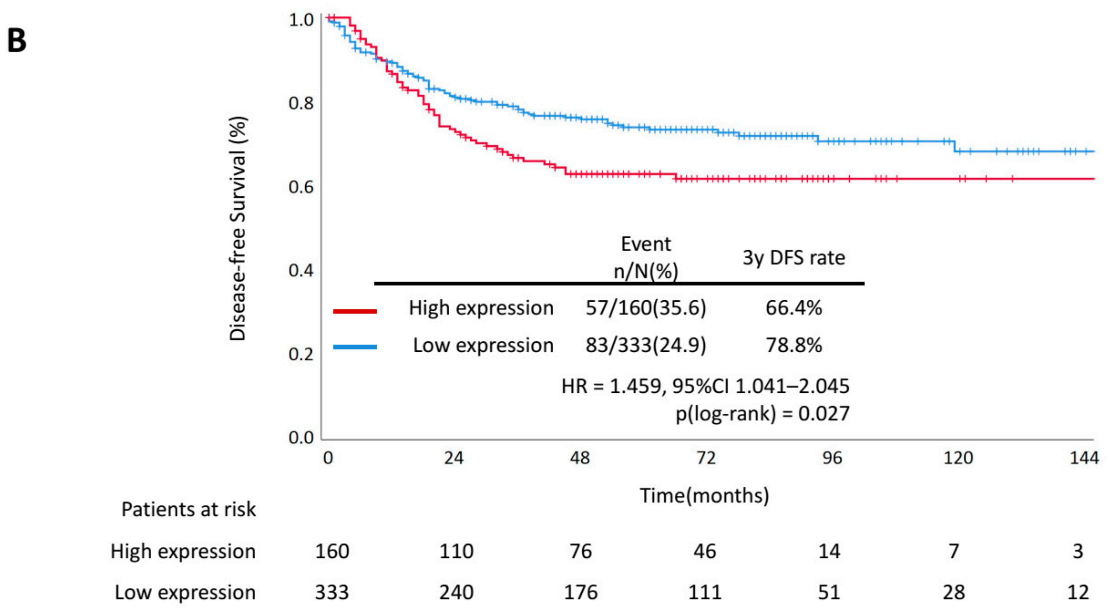


**Figure S1.** Kaplan-Meier curves of DFS stratified by CUL9 expression in stage I-III patients. (**A**) The primary cohort; (**B**) The validation cohort. HR, Hazard ratio; CI, Confidence interval.

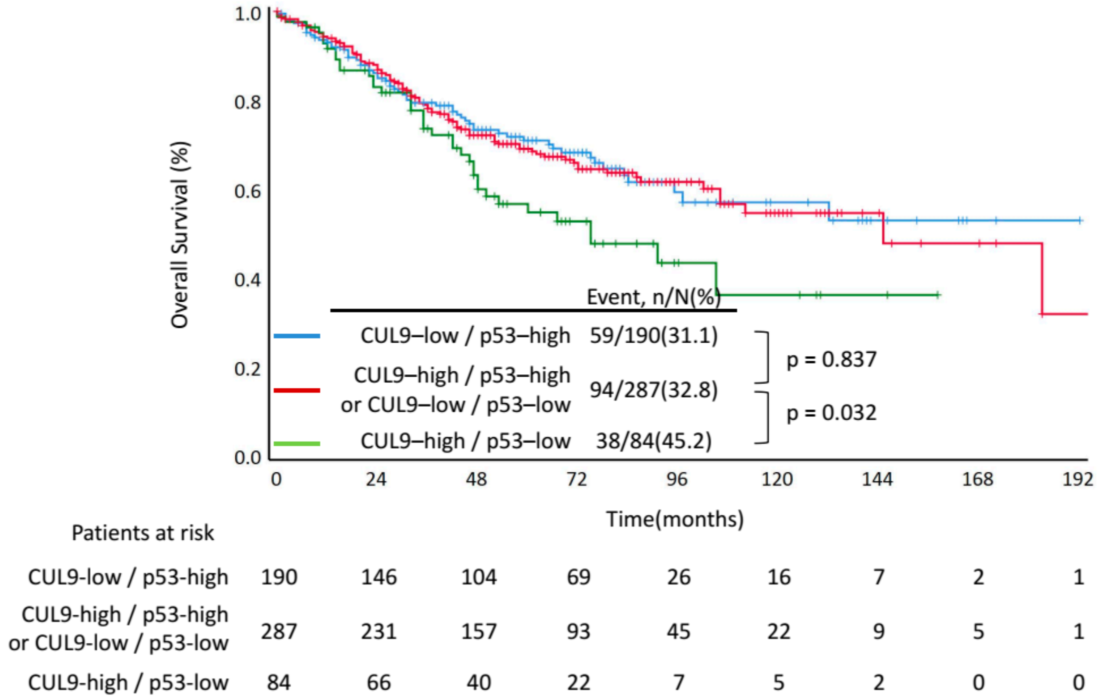


**Figure S2.** Kaplan-Meier curves of OS stratified by CUL9 and p53 expression in the validation cohort. High and low p53 expression groups were divided according to the median mRNA expression of p53. HR, Hazard ratio; CI, Confidence interval.

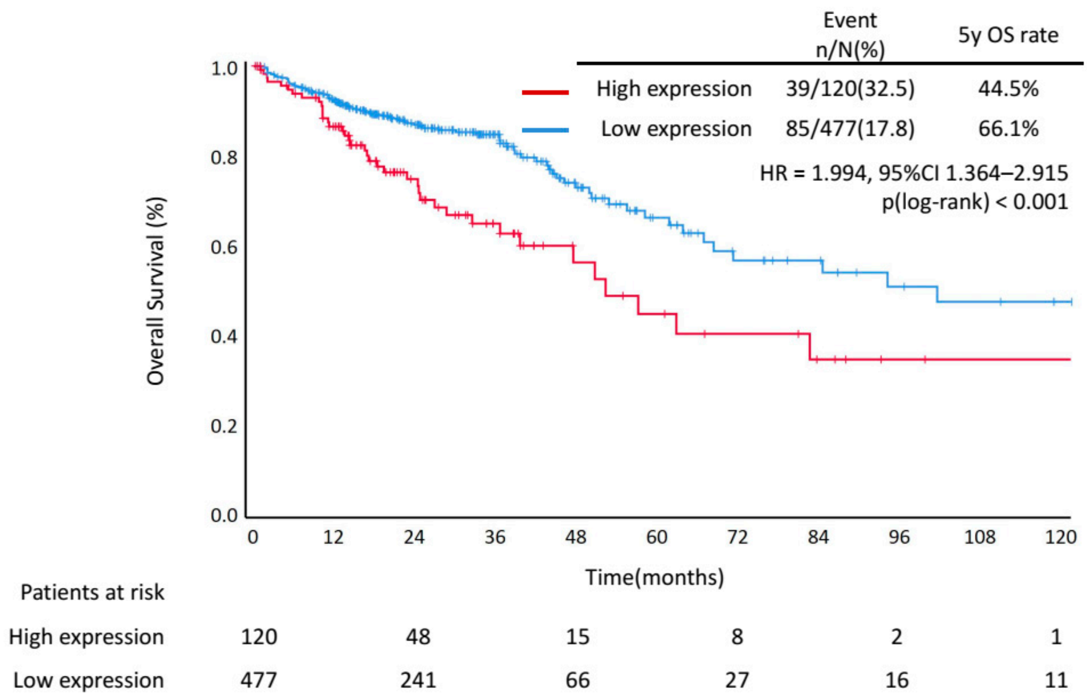


**Figure S3.** Kaplan-Meier curves of OS stratified by CUL9 expression in the cohort from TCGA database. High and low expression groups were divided according to the cutoff value of FPKM of RNA sequencing. The cutoff value represented the best prognostic efficacy on OS.HR, Hazard ratio; CI, Confidence interval.

**Table S1.** Summary of RAS and BRAF mutations tested by ARMS.

| Gene | Exon | Mutation Loci* |
|---|---|---|
| KRAS | 2 | G12S, G12D |
| | 2 | G12C, G12R, G12V, G12A, G13C |
| | 2 | G13D |
| | 3 | Q61H, Q61L, Q61R |
| | 4 | K117N, A146T, A146V, A146P |
| NRAS | 2 | G12S, G12D |
| | 2 | G13D |
| | 2 | G12A, G12V, G12C, G13R, G13V |
| | 3 | Q61K, Q61H, Q61L, Q61R |
| | 4 | A146T |
| BRAF | 15 | V600E |

*Mutations in the same table cell were tested in one PCR tube and are not distinguished respectively.

**Table S2.** Uni- and multivariate analysis of disease-free survival in the primary cohort.

| | **Univariate analysis** | | | **Multivariate analysis*** | | |
|---|---|---|---|---|---|---|
| | **HR** | **95%CI** | ***P* Value** | HR | **95%CI** | ***P* Value** |
| Age, years | | | 0.970 | | | |
| <60 | 1 | - | | | | |
| ≥60 | 0.938 | 0.696–1.264 | | | | |
| Gender | | | 0.862 | | | |
| Female | 1 | - | | | | |
| Male | 0.974 | 0.721–1.314 | | | | |
| Pre-operative CEA , ng/mL | | | **0.021** | | | 0.388 |

| | | | | | | |
|---|---|---|---|---|---|---|
| ≤5 | 1 | - | | 1 | - | |
| >5 | 1.422 | 1.054–1.919 | | 1.147 | 0.840–1.566 | |
| Tumor location | | | **0.039** | | | 0.065 |
| Right-sided | 1 | - | | 1 | | |
| Left-sided | 0.727 | 0.537–0.984 | | 0.730 | 0.522–1.020 | |
| Histological grade | | | **0.014** | | | 0.160 |
| Low/Undifferentiated | 1 | - | | 1 | - | |
| Well/Moderate | 0.677 | 0.495–0.925 | | 0.796 | 0.578–1.095 | |
| T stage | | | | | | |
| T1–2 | 1 | - | | 1 | - | |
| T3 | 2.081 | 1.220–3.549 | **0.007** | 1.594 | 0.921–2.759 | 0.096 |
| T4 | 2.376 | 1.383–4.082 | **0.002** | 1.605 | 0.899–2.865 | 0.110 |
| N stage | | | | | | |
| N0 | 1 | - | | 1 | - | |
| N1 | 1.514 | 1.077–2.130 | **0.017** | 1.298 | 0.911–1.850 | 0.149 |
| N2 | 2.759 | 1.842–4.132 | **<0.001** | 2.307 | 1.525–3.490 | **<0.001** |
| Vascular invasion | | | 0.131 | | | |
| No | 1 | - | | | | |
| Yes | 1.431 | 0.898–2.281 | | | | |
| Perineural invasion | | | **0.014** | | | 0.268 |
| No | 1 | - | | 1 | - | |
| Yes | 2.031 | 1.154–3.576 | | 1.399 | 0.772–2.535 | |
| Tumor deposits† | | | **<0.001** | | | |
| No | 1 | - | | | | |
| Yes | 2.172 | 1.547–3.049 | | | | |
| RAS status | | | 0.428 | | | |
| Wild-type | 1 | - | | | | |
| Mutant | 1.128 | 0.837–1.522 | | | | |
| BRAF status | | | **<0.001** | | | **<0.001** |
| Wild-type | 1 | - | | 1 | - | |
| Mutant | 3.371 | 2.172–5.232 | | 2.847 | 1.816–4.461 | |
| MMR status | | | 0.732 | | | |
| dMMR | 1 | - | | | | |
| pMMR | 0.916 | 0.556–1.511 | | | | |
| CUL9 expression | | | **0.002** | | | **0.004** |
| Low | 1 | - | | | | |
| High | 1.596 | 1.182–2.156 | | 1.570 | 1.159–2.128 | |

Abbreviation: CEA, Carcinoembryonic antigen; MMR, mismatch repair; pMMR, proficient mismatch repair; dMMR, deficient mismatch repair; HR, Hazard ratio; CI, Confidence interval. *: Multivariate analysis included those variates with p<0.10 in univariate analysis. †: N1c stage was defined as that no regional lymph nodes are positive, but tumor deposits are detected. Therefore, tumor deposit was not included in multivariate analysis because of the correlation with N stage.

**Table S3.** Correlation between baseline characteristics and CUL9 expression in the validation cohort.

| | **Total (*N*=565)** | **High expression group (*N*=187)** | **Low expression group (*N*=378)** | ***P* Value** |
|---|---|---|---|---|
| Age, years, n (%) | | | | 0.866 |
| >=60 | 415(73.5%) | 140(74.8%) | 275(72.8%) | |
| <60 | 150(26.5%) | 47(25.2%) | 103(27.2%) | |
| Gender, n (%) | | | | 0.624 |
| Male | 309(54.7%) | 105(56.1%) | 204(54.0%) | |
| Female | 256(45.3%) | 82(43.9%) | 174(46.0%) | |
| Tumor location, n (%) | | | | 0.741 |

| | | | | |
|---|---|---|---|---|
| Right-sided | 223(39.5%) | 72(38.5%) | 151(39.9%) | |
| Left sided | 342(60.5%) | 115(61.5%) | 227(60.1%) | |
| T stage, n (%) | | | | 0.545 |
| T1-T2 | 59(10.4%) | 18(9.6%) | 41(10.8%) | |
| T3 | 367(65.0%) | 124(66.3%) | 243(64.3%) | |
| T4 | 119(21.1%) | 34(18.2%) | 85(22.5%) | |
| Unknown | 20(3.5%) | 11(5.9%) | 9(2.4%) | |
| N stage, n (%) | | | | 0.090 |
| N1-2 | 244(43.2%) | 88(47.1%) | 156(41.3%) | |
| N0 | 301(53.3%) | 88(47.1%) | 213(56.3%) | |
| Unknown | 20(3.5%) | 11(5.9%) | 9(2.4%) | |
| TNM stage, n (%) | | | | 0.437 |
| I | 33(5.8%) | 9(4.8%) | 24(6.3%) | |
| II | 264(46.7%) | 81(43.3%) | 183(48.4%) | |
| III | 205(36.3%) | 76(40.6%) | 129(34.1%) | |
| IV | 60(10.6%) | 19(10.2%) | 41(10.8%) | |
| Unknown | 3(0.5%) | 2(0.1%) | 1(0.3%) | |
| KRAS status, n (%) | | | | 0.517 |
| Wild-type | 328(58.1%) | 104(55.6%) | 224(59.3%) | |
| Mutant | 216(38.2%) | 74(39.6%) | 142(37.6%) | |
| Unknown | 21(3.7%) | 9(4.8%) | 12(3.2%) | |
| BRAF V600E status, n (%) | | | | 0.191 |
| Wild-type | 460(81.4%) | 160(85.6%) | 300(79.4%) | |
| Mutant | 51(9.0%) | 14(7.5%) | 37(9.8%) | |
| Unknown | 54(9.6%) | 13(7.0%) | 41(10.8%) | |
| MMR status, n (%) | | | | 0.094 |
| pMMR | 443(78.4%) | 154(82.4%) | 289(76.5%) | |
| dMMR | 75(13.3%) | 24(12.8%) | 51(13.5%) | |
| Unknown | 47(8.3%) | 9(4.8%) | 38(10.1%) | |

Abbreviation: MMR, mismatch repair; pMMR, proficient mismatch repair; dMMR, deficient mismatch repair.

**Table S4.** Summary of adjuvant chemotherapy in high-risk stage II and stage III patients from the primary cohort.

| Adjuvant Chemotherapy | Total (*N*=564) | High-Risk Stage II (*N*=263) | Stage III (*N*=301) |
|---|---|---|---|
| Chemotherapy regimens | | | |
| FOLFOX, n(%) | 405(71.7%) | 160(60.8%) | 245(81.4%) |
| CapOX, n(%) | 159(28.3%) | 103(39.2%) | 56(18.6%) |
| Groups according to finished cycles | | | |
| Full dose group, n(%) (at least 75% of planned cycles) | 384(68.0%) | 181(68.8%) | 203(67.4%) |
| Cumulative dose, % | 90.9% | 89.4% | 92.2% |
| Reduced dose group, n(%) (25% to 75% of planned cycles) | 129(22.8%) | 56(21.3%) | 73(24.3%) |
| Cumulative dose, % | 59.0% | 60.8% | 57.6% |
| Planned 3 months regimen* | 40(7.1%) | 19(7.2%) | 21(7.0%) |
| Adverse events† | 62(11.0%) | 24(9.1%) | 34(11.3%) |
| Withdrew | 27(4.8%) | 13(4.9%) | 14(4.6%) |
| Low dose group, n(%) (at most 25% of planned cycles) | 51(9.0%) | 26(9.9%) | 25(8.3%) |
| Cumulative dose, % | 8.8% | 7.1% | 10.7% |
| Refused | 38(6.7%) | 20(7.6%) | 18(6.0%) |
| Adverse events‡ | 13(2.3%) | 6(2.3%) | 7(2.3%) |

*: Three months regimen was considered for patients with elder age, cirrhosis, diabetic peripheral neuropathy, and other conditions. †: Adverse events that result in dose reduction mainly included

hand-foot syndrome, neutropenia, and thrombocytopenia. ‡: Adverse events that result in termination of chemotherapy mainly included allergic reaction and neutropenia.

**Table S5.** Efficacy of adjuvant chemotherapy stratified by CUL9 expression in high risk stage II and stage III patients from the primary cohort.

| | **Total (*N*=513)** | | **High Expression Group (*N*=166)** | | **Low Expression Group (*N*=347)** | |
|---|---|---|---|---|---|---|
| | Full dose group* (N=384) | Reduced dose group† (N=129) | Full dose group (N=120) | Reduced dose group (N=46) | Full dose group (N=263) | Reduced dose group (N=84) |
| Recurrence rate | 22.9% | 29.5% | 25.8% | 47.8% | 21.6% | 19.3% |
| OR | 0.712 | | 0.380 | | 1.153 | |
| 95% CI | 0.455-1.113 | | 0.187-0.722 | | 0.621-2.142 | |
| p for chi-square test | 0.135 | | **0.007** | | 0.652 | |
| p for correlation test | | | **0.021** | | | |
| Disease-free survival | | | | | | |
| 3y-DFS rate | 78.1% | 71.2% | 76.0% | 55.3% | 78.5% | 80.6% |
| HR | 0.732 | | 0.477 | | 1.092 | |
| 95% CI | 0.500-1.072 | | 0.276-0.825 | | 0.527-1.903 | |
| p for log-rank test | 0.106 | | **0.006** | | 0.754 | |
| p for correlation test | | | **0.034** | | | |

Abbreviation: OR, Odd ratio; HR, Hazard ratio; CI, Confidence interval. *: Full dose group consists of patients who had finished at least 75% of planned cycles. †: Reduced dose group consists of patients who had finished 25% to 75% of planned cycles.
